# Acylated ghrelin levels were associated with depressive status, physical quality of life, endoscopic findings based on Kyoto classification in Japan

**DOI:** 10.3164/jcbn.18-111

**Published:** 2019-05-18

**Authors:** Shuhei Agawa, Seiji Futagami, Hiroshi Yamawaki, Go Ikeda, Hiroto Noda, Kumiko Kirita, Kazutoshi Higuchi, Makoto Murakami, Yasuhiro Kodaka, Nobue Ueki, Takashi Akamizu, Katsuhiko Iwakiri

**Affiliations:** 1 Department of Internal Medicine, Division of Gastroenterology, Nippon Medical School, 1-1-5 Sendagi, Bunkyo-ku, Tokyo 113-8602, Japan; 2 The First Department of Medicine, Wakayama Medical University, 811-1 Kimiidera, Wakayama 641-8509, Japan

**Keywords:** functional dyspepsia, gastric motility, acylated ghrelin, Kyoto classification, SRQ-D

## Abstract

Background and Aims: To determine whether serum acylated ghrelin levels were associated with anxiety, clinical symptoms, depressive status, quality of life, gastric motility and endoscopic findings based on Kyoto classification in functional dyspepsia (FD) patients. Methods: We enrolled three groups, FD patients (*n* = 15) with high levels of acylated ghrelin, FD patients (*n* = 33) with normal levels of acylated ghrelin and FD patients (*n* = 35) with low levels of acylated ghrelin. There was no significant differences in the positivity of *Helicobacter pylori* infection among the three groups. Clinical symptoms were evaluated by Gastrointestinal Symptom Rating Scale (GSRS) and FD symptoms based on Rome III classification. Acylated ghrelin levels were measured by ELISA methods. Depressive status, anxiety, sleep disturbance were respectively asscessed by Self-rating questionnaire for depression (SRQ-D) score, STAI-state/-trait, Pittsburgh sleep quality index (PSQI) scores. Endoscopic findings were evaluated based on Kyoto classification. Results: Body Mass Index (BMI) in FD patients with low levels of acylated ghrelin was significantly higher (*p*<0.001 and *p* = 0.008, respectively) compared to those in FD patients with high and normal levels of acylated ghrelin. SRQ-D scores in FD patients with low levels of acylated ghrelin was significantly lower (*p* = 0.008 and *p*<0.001, respectively) compared to those in FD patients with high and normal levels of acylated ghrelin. Scoring of gastric atrophy, intestinal metaplasia, xanthoma and mucus based on Kyoto classification in FD patients with low levels of acylated ghrelin were significantly higher (*p*<0.001, *p* = 0.0077, *p* = 0.036 and *p* = 0.0063, respectively) compared to those in FD patients with more than low levels of acylated ghrelin. Conclusion: Acylated ghrelin levels were associated with BMI, depressive status, and endoscopic findings based on Kyoto classification in FD patients.

## Introduction

Ghrelin, a 28-amino acid, novel appetite-stimulating peptide produced predominantly by the stomach, is thought to be a strong growth hormone releaser.^(1)^ Since ghrelin plays a role in the long-term as well as short-term regulation of feeding,^(2)^ in human studies, the treatment with ghrelin increases food intake and the sensation of hunger compared to the saline infusion alone.^(3)^ In addition, ghrelin is also an immunomodulatory hormone, providing new perspectives for its relevance in metabolic diseases^(4,5)^ and acylated ghrelin has been reported to suppresses the cytokine production response to Lipopolysaccharide (LPS).^(6)^ The plasma ghrelin levels raise during fasting and falls quickly after meals.^(7)^ Since ghrelin was mainly produced and acylated in the stomach, Ulasoglu *et al.*^(8)^ reported that circulating ghrelin levels decreased in *Helicobacter pylori* (*H. pylori*) eradicated subjects. However, a systematic review using 25 studies addressed that eradicating *H. pylori* did not influence circulating ghrelin levels.^(9)^ Moreover, there was no available data about the relationship between endoscopic findings and acylated ghrelin levels in functional dyspepsia (FD) patients.

In Japan, the new Kyoto Global Consensus Meeting on *H. pylori* gastritis proposed that the risk of *H. pylori*-infected gastritis should be determined according to the extent of gastric atrophy, gastric inflammation and intestinal metaplasia.^(10)^ Kyoto classification was reported to support diagnostic and management strategies aimed at gastric cancer prevention.^(11,12)^ Considering previous reports,^(11–14)^ it is very important to evaluate the accumulated risk of *H. pylori*-infected gastritis using the endoscopic, histological and topographical assessments provided in Kyoto Global Consensus Meeting. Kyoto classification were evaluated by the extent of intestinal metaplasia, gastric atrophy and gastric inflammation after *H. pylori* eradication therapy.

We have previously reported the acylated ghrelin levels in FD patients, Nonerosive Reflux Disease (NERD) patients and healthy volunteers.^(15)^ In the previous report,^(15)^ acylated ghrelin levels in certain group in Postprandial Distress Syndrome (PDS) patients have a negative correlation to gastric emptying. Recently, serum acylated ghrelin levels have been reported to be associated with gastric emptying, anxiety,^(16)^ depressive status^(17)^ and intestinal metaplasia.^(18)^ In this study, we have first investigated whether serum acylated ghrelin levels were associated with anxiety, clinical symptoms, depressive status, quality of life, gastric motility and endoscopic findings based on Kyoto classification in FD patients.

## Materials and Methods

### Patients

One hundred-thirteen consecutive patients presenting with typical symptoms of FD were enrolled after upper gastrointestinal endoscopy and abdominal ultrasonography. Patients were diagnosed according to Rome III criteria.^(19)^ We enrolled three groups, FD patients (*n* = 15) with high levels of serum acylated ghrelin, FD patients (*n* = 33) with normal levels of serum acylated ghrelin and FD patients (*n* = 35) with low levels of serum acylated ghrelin. High and low levels of acylated ghrelin level were defined as 3 standard distribution (SD) more than or less than mean value of ghrelin levels in healthy volunteers. Exclusion criteria included severe heart disease, renal or pulmonary failure, liver cirrhosis, severe systemic illness and history of malignant disease. Patients with previous gastroduodenal surgery, duodenal ulcer scar, diabetes mellitus, and recent use of non-steroid anti-inflammatory drugs (NSAIDs), proton pump inhibitors (PPIs) or anticoagulants at endoscopy were also excluded. *H. pylori* infection was determined by both the ^13^C-urea breath test and by histological identification. Written informed consent was obtained from all subjects prior to undergoing upper gastrointestinal endoscopy and abdominal ultra-sonography for evaluation of their dyspeptic symptoms. The study protocol was approved by the Ethics Review Committee of Nippon Medical School Hospital.

### Clinical symptoms

Clinical symptoms of FD were evaluated according to the Rome III criteria.^(19)^ Clinical symptoms must have involved at least one of the following: early satiation, bothersome postprandial fullness, epigastric pain, or epigastric burning. FD symptoms were evaluated as follows: 0, none; 1, very mild; 2, mild; 3, moderate; 4, severe; 5, very severe. Clinical symptoms were evaluated with the Gastrointestinal Symptom Rating Scale (GSRS).^(20)^ The GSRS is composed of 15 items that generate 5 components including gastroesophageal reflux, abdominal pain, indigestion, diarrhea and constipation. Each item was rated according to severity on a scale of 1 (no discomfort at all) to 7 (very severe discomfort). We used the mean score of the GSRS and the 15 GI symptoms of the GSRS for the evaluation of dyspeptic symptoms.

### Assessment of endoscopic appearance

Endoscopic analyses were conducted. All enrolled patients were examined endoscopically. The overall findings included diffuse redness, patchy redness, spotty redness, enlarged fold, atrophic fold, visibility of vascular pattern, intestinal metaplasia, mucosal swelling, swelling of area gastrica, nodularity of gastric mucosa, kammrotung, xanthoma, mucus, fundic gland polyp, regular arrangement of collecting venules (RAC) in angle, flat erosion, raised erosion and hematin. These findings were scored for each patient as follows: 0, absent; and 1, present for each patient based on the modified previous study.^(12)^ In addition, to evaluate the extent of gastric atrophy, gastric inflammation and intestinal metaplasia, the sum of scores of diffuse redness, enlarged folds, intestinal metaplasia and visibility of vascularity based on Kyoto classification, each factor were estimated as follows: score 0, their distribution could not be observed endoscopically; score 1, their distribution were localized to the gastric antrum; score 2, their distribution were localized to the gastric body.^(11,12)^ To standardize endoscopic findings, participating endoscopists were given a detailed description of each type of endoscopic finding that was to be analyzed in this study, including representative photographs. All endoscopic findings were retrospectively evaluated by four principal investigators (SF, HN, GI, HY and SA). When opinions differed among endoscopists, a final judgement was arrived at by consensus following a discussion of each individual case.

### State-trait anxiety inventory (STAI)

The STAI is a well-validated 40-item self-reported questionnaire to evaluate degree of anxiety.^(21)^ State of anxiety reflects a “transitory emotional state or condition of the human organism that is characterized by subjective, consciously perceived feelings of tension and apprehension, and heightened autonomic nervous system activity”. State of anxiety may fluctuate over time and can vary in intensity. In contrast, trait of anxiety denotes “relatively stable individual differences in anxiety proneness”.

### Self-rating questionnaire for depression (SRQ-D)

Status of depression was evaluated by Self-Rating Questionnaire for Depression. The SRQ-D comprises 18 items, which are rated on a 4-point scale (0 = ‘no’, 1 = ‘sometimes’, 2 = ‘frequently’, and 3 = ‘always’). Among these 18 questions, 6 non-relevant questions are interspersed. According to the diagnostic criteria for depression, subjects with scores of 9 points or less are considered to be normal; those with scores of 10–15 points as borderline; those with scores of 16 points or more as mild depression. In this study, we determined the patients with SRQ-D scores of 16 points or more as having depressive symptoms.^(22)^

### Health-related quality of life (HRQOL)

The Social Functioning-8 (SF-8) test was used to measure health-related quality of life according to the *Manual of the SF-8 Japanese Version*.^(23)^ The SF-8 scores 8 domains, including general health, physical functioning, mental health and role-emotional that are then combined with a physical component summary (PCS) and a mental component summary (MCS) to arrive at a composite score. A score <50 thus indicates impaired quality of life (QOL) with lower scores considered to indicate greater damage to QOL.

### Pittsburgh sleep quality index (PSQI)

Sleep quality and sleep duration were evaluated by a Japanese version of the Pittsburgh Sleep Quality Index (PSQI) questionnaire.^(24)^ The score of each component ranges from 0 to 3, reflecting severity of symptoms and the sum of the seven component scores provide a global PSQI score that ranges from 0 to 21. Higher scores indicate poorer sleep.^(24,25)^ A cut-off score >5.5 has a sensitivity of 80.0–85.7% for various patient groups, and a specificity of 86.6% for control subjects in the Japanese version of the PSQI.^(24)^

### Measurement of plasma ghrelin in FD patients

We measured plasma ghrelin levels in FD patients. Blood samples were obtained after an overnight fast of >12 h, immediately transferred to chilled polypropylene tubes containing Na_2_EDTA and aprotinin, then centrifuged at 4°C. One tenth of the volume of 1NHCl was immediately added to the separated plasma. The acylated and des-acylated forms of ghrelin were measured using commercially available ELISA kits according to the manufacturer’s instructions (Active Ghrelin ELISA Kit and Desacly-Ghrelin ELISA Kit, Mitsubishi Kagaku Iatron Inc., Tokyo, Japan). The intra- and inter-assay coefficients of variation (CV) were 6.5 and 9.8% for acylated ghrelin, and 3.7 and 8.1% for des-acylated ghrelin.

### Measurement of gastric emptying

Sodium acetate (water soluble; ^13^C-acetate) for emptying of liquids was used as a tracer (Cambridge Isotope Laboratories; Tewksbury, MA). The liquid test meal consisted of 100 mg of ^13^C-acetate dissolved in 200 ml of a liquid meal (Racol, 1 ml/kcal; Otsuka Pharmacia Company, Tokyo, Japan). Breath samples were collected 0 s, 10 s, 5 min, 10 min, 15 min, 20 min, 30 min, 40 min, 50 min, 60 min, 75 min and 90 min after ingestion of the test meal at 10:00 a.m. The subject’s own production of 300 mM CO_2_ per m^2^ body surface and per hour were set as default. We used an Integrated Software Solutions program to calculate the half gastric emptying time (T_1/2_) and the lag phase (Tmax; min) as the point of maximum gastric emptying according to Hellmig *et al.*^(26)^ The half gastric emptying time (T_1/2_) represents the time when 50% of the initial gastric content was emptied. Tmax value greater than 60 min, representing the mean Tmax in healthy volunteers plus SD, was defined to represent relative disturbances in gastric emptying according to the diagnostic criteria of the Japan Society of Smooth Muscle Research and our study.^(15,27)^

### Data analysis

The time plot of pulmonary [^13^CO_2_] excretion (%dose/h) was fitted to the function: (%dose/h) = m × k × β × e^−kt^ × (1 – e^−kt^)^β^^−1^

where “m” is the cumulative [^13^CO_2_] recovery at the infinite time, “t” is in hours and “k” and “β” are regression-estimated constants.

(Cumulative %dose) = m × (1 – e^−kt^)^β^

AUC: area under the curve

AUC_5_ = m × (1 – e^−k^^ × ^^0.08^)^β^ [T: 5 min = 0.08 h]

AUC_15_ = m × (1 – e^−k^^ × ^^0.25^)^β^ [T: 15 min = 0.25 h]

AUC_30_ = m × (1 – e^−k^^ × ^^0.5^)^β^ [T: 30 min = 0.5 h]

AUC_60_ = m × (1 – e^−k^^ × ^^1.0^)^β^ [T: 60 min = 1.0 h]

AUC_90_ = m × (1 – e^−k^^ × ^^1.5^)^β^ [T: 90 min = 1.5 h]

We determined the area under the curve at 5 min (AUC_5_) and at 15 min (AUC_15_) values as markers of the early phase of gastric emptying based on previous studies^(28,29)^ AUC_5_ values of >17.4 and AUC_15_ values of >39.6, representing the mean AUC value of healthy volunteers plus 2SD, were defined to represent disturbances in the early phase of gastric emptying.

## Results

### Characteristics of the enrolled patients

**Fig. 1:**
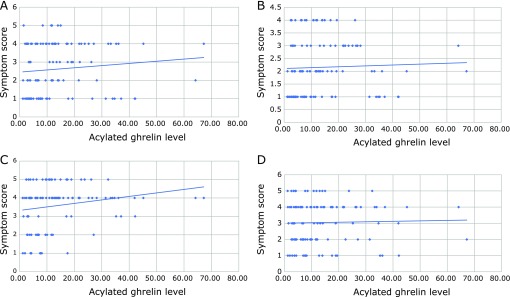
There were not significant relationships between clinical symptoms and acylated ghrelin levels in FD patients. (A) The relationship between symptom score of epigastric pain and acylated ghrelin levels. (B) The relationship between symptom score of epigastric burning and acylated ghrelin levels. (C) The relationship between symptom score of postprandial fullness and acylated ghrelin levels. (D) The relationship between symptom score of early satiety and acylated ghrelin levels.

We enrolled three groups, FD patients (*n* = 15) with high levels of serum acylated ghrelin, FD patients (*n* = 33) with normal levels of serum acylated ghrelin and FD patients (*n* = 35) with low levels of serum acylated ghrelin. High and low levels of acylated ghrelin were defined as 3 SD more than or less than mean value of ghrelin levels in healthy volunteers. There were no significant differences in sex and Gastrointestinal Symptom Rating Scale (GSRS) among three FD groups (Table 1). Furthermore, FD symptoms such as epigastric pain, epigastric burning, postprandial fullness and early satiety were not significantly associated with acylated ghrelin levels in FD patients (Fig. 1A–D). Body Mass Index (BMI) in FD patients with low levels of acylated ghrelin was significantly higher (*p*<0.001 and *p* = 0.008, respectively) compared to those in FD patients with higher and normal levels of acylated ghrelin (Table 1). There were no significant differences in the positivity of *H. pylori* infection (12.5%, 19.2% and 25%) among three groups.

### Comparison of anxiety, depression, quality of life and sleep disturbances among three groups

Since ghrelin levels have been reported to be associated with anxiety and depression, we tried to clarify whether serum acylated ghrelin levels affected STAI, SRQ-D and PSQI scores in FD patients. There were no significant differences in STAI-state scores among three groups (Table 2). However, SRQ-D scores in FD patients with high and normal levels of acylated ghrelin were significantly (*p* = 0.040 and *p* = 0.0035, respectively) high compared to those in FD patients with low levels of acylated ghrelin (Table 2). In addition, physical component summary (PCS) in FD patients with high and normal levels of acylated ghrelin were significantly (*p* = 0.008 and *p*<0.001, respectively) disturbed compared to that in FD patients with low levels of acylated ghrelin (Table 2). However, there was no significant differences in mental component summary (MCS) among three groups. Moreover, we tried to clarify whether acylated ghrelin levels in FD patients was associated with the sleep disturbances. PSQI score of FD patients with high levels of acylated ghrelin was significantly lower (*p* = 0.0076) compared to that in FD patients with normal levels of acylated ghrelin. In addition, PSQI score of FD patients with normal levels of acylated ghrelin was significantly higher (*p* = 0.002) compared to that in FD patients with low levels of acylated ghrelin. There were no significant differences in PSQI values among three groups (Table 2).

### Comparison of gastric emptying with acylated ghrelin levels in FD patients

Since ghrelin have been studied to affect gastric motility, we tried to clarity whether serum acylated ghrelin levels affected Tmax and T_1/2_ values as the marker of gastric emptying in FD patients. There were no significant differences in Tmax and T_1/2_ values among three groups, albeit Tmax and T_1/2_ values in FD patients with low levels of acylated ghrelin have relatively higher tendency (*p* = 0.41 and *p* = 0.38, respectively) to those in FD patients with high acylated ghrelin levels (Table 3).

Then, we tried to clarify whether acylated ghrelin levels improved AUC_5_ and AUC_15_ values as a marker of early gastric emptying in FD patients. There were no significant differences in AUC_5_ and AUC_15_ values among these groups, albeit AUC_5_ and AUC_15_ values in FD patients with high levels of acylated ghrelin were relatively (*p* = 0.054 and *p* = 0.076, respectively) disturbed compared to that in FD patients with low levels of acylated ghrelin (Table 3).

### Relationship between endscopic findings based on Kyoto classification and acylated ghrelin levels in FD patients

Since previous studies have been demonstrated that serum ghrelin levels were associated with gastric atrophy and advances in intestinal metaplasia, we tried to clarify whether there were significant relationships between acylated ghrelin levels and endoscopic findings based on Kyoto classification. We compared endoscopic findings based on Kyoto classification with FD patients with low levels of acylated ghrelin. Interestingly, scoring of gastric atrophy, intestinal metaplasia, xanthoma and mucus in FD patients with low levels of acylated ghrelin were significantly higher (*p*<0.001, *p* = 0.0077, *p* = 0.036 and *p* = 0.0063, respectively) compared to those in FD patients with more than low levels of acylated ghrelin (Table 4).

## Discussion

The major findings of this clinical study on the associations between several factors and acylated ghrelin levels in FD patients are following as: 1) BMI in FD patients with high levels of acylated ghrelin was significantly lower compared to that in FD patients with low levels of acylated ghrelin. 2) SRQ-D scores in FD patients with high levels of acylated ghrelin was significantly higher compared to that in FD patients with low levels of acylated ghrelin. 3) PCS in FD patients with high levels of acylated ghrelin was significantly lower compared to that in FD patients with low levels of acylated ghrelin. 4) Scoring of gastric atrophy, intestinal metaplasia, xanthoma and mucus based on Kyoto classification in FD patients with low levels of acylated ghrelin was significantly higher compared to those in FD patients with more than low levels of acylated ghrelin.

Algul *et al.*^(30)^ have reported that the acylated ghrelin levels were found to be significantly higher in severe major depression disorders as compared to control group. In our data, SRQ-D score in FD patients with high levels of acylated ghrelin was significantly higher compared to that in low levels of acylated ghrelin. Thus, our data have first demonstrated that acylated ghrelin levels were associated with depressive state in FD patients as well as the patients with major depression disorders. In contrast, there were not significant differences in anxiety among three groups as described in Table 2. Lutter *et al.*^(17)^ have studied that ghrelin has anxiolytic-like effects and that ghrelin signaling is reguired for the anxiolytic-like effects of caloric restriction. Further studies will be needed to determine the reason why acylated ghrelin significantly affect anxiety in FD patients. In our data, acylated ghrelin levels did not affect GSRS and each FD symptoms such as epigastric pain, epigastric burning, abdominal fullness and early satiety. In addition, ghrelin has also been shown to be an critical player in the regulation of numerous central system, such as sleep, mood and reward.^(16,31)^ Kluge *et al.*^(32)^ have addressed that elderly males administered ghrelin were subsequently characterized by an increased proportion of stage 2 and SWS sleep and decreased stage1 and Rapid eye movement (REM) sleep. However, in our study, there was no significant associations between PSQI scores and serum acylated ghrelin levels in FD patients. Totally, in our data, low levels of acylated ghrelin was significantly associated with physical quality of life in FD patients. Measurement of acylated ghrelin levels might be a useful tool to determine the cessation of therapy for FD patients. However, several studies suggest that the relationship between plasma ghrelin levels and FD patients remain uncertain.^(15,28,33)^ Further studies will be warrented to evaluate same trials in multi-center, other races and larger sample size.

In previous our data, Leu 72 Met 408 polymorphism of the ghrelin gene was not associated with gastric emptying in the patients with FD.^(34)^ In our study, there was no significant association between gastric motility and acylated ghrelin levels in FD patients. Considering that gastric motility is affected by various gut hormones such as motilin, gherelin, cholecystokinin, glucagon-like peptide (GLP) 1 and peptide YY,^(35)^ further studies will be needed to measure other gut hormones such as GLP-1. Moreover, Lee *et al.*^(29)^ have reported that gastric flow into the duodenum including gastric acid inhibits gastric accommodation to a meal and contributes to postprandial symptom. These early gastric emptying has been reported to be very critical issues for pathophysiology in FD patients.^(34,36)^ Therefore, in this study, we tried to determine whether there was a significant relationship between early phase of gastric emptying and acylated ghrelin levels in FD patients. Considering there was a relative associations between early gastric emptying and acylated ghrelin levels, high levels of acylated ghrelin may partly play important roles in the disturbance of early gastric emptying such as AUC_5_ and AUC_15_ values.

In this study, we tried to determine whether there were significant relationships between acylated ghrelin levels and each endoscopic findings based on Kyoto classification. The advanced extent of gastric atrophy and intestinal metaplasia were significantly associated with the levels of acylated ghrelin. Since ghrelin-producing cells are distributed in the gastric mucosa, the extent of gastric atrophy may be related to the reduction of the production of ghrelin. Suzuki *et al.*^(37)^ have reported that the plasma ghrelin levels and are decreased in association with advancing gastric mucosal atrophy. Gao *et al.*^(38)^ have also demonstrated that plasma gherelin levels were reduced in atrophic gastritis compared to those in healthy subjects. In contrast, Kim *et al.*^(18)^ have demonstracted that there was no significant association between ghrelin levels and gastric atrophy. Kim *et al.*^(18)^ have reported that plasma ghrelin level are associated with intestinal metaplasia in elderly patients with functional dyspepsia. Further studies will be needed whether acylated ghrelin levels may negatively contributed to the expression or the extent of intestinal metaplasia.

Taken together, in this study, plasma acylated ghrelin levels were associated with several factors including endoscopic findings based on Kyoto classification in FD patiens, albeit further studies will be needed to clarify the precise roles in ghrelin for the etiology of FD patients.

## Figures and Tables

**Table 1:** Characteristics of the enrolled patients

	FD patients (*n* = 113)	FD patients with high levels of acylated ghrelin (*n* = 15)	FD patients with normal levels of acylated ghrelin (*n* = 33)	FD patients with low levels of acylated ghrelin (*n* = 35)
Age	16–90	24–80	30–90	47–84
Sex (F/M)	66/47	12/3	20/13	16/19
BMI	21.7 ± 3.17	19.8 ± 0.58*	21.3 ± 0.51**	23.3 ± 0.53
GSRS	2.55 ± 0.94	2.67 ± 0.29	2.63 ± 0.16	2.35 ± 0.15
The positivity of *H. pylori* infection	22.4%	12.5%	19.2%	25%

*p<0.001, vs FD patients with low levels of acylated ghrelin; **p = 0.008, vs FD patients with low levels of acylated ghrelin; GSRS, Gastrointestinal Symptom Rating Scale.

**Table 2:** Comparison of anxiety, depression, quality of life and sleep disturbances among three groups

	FD patients with high levels of acylated ghrelin (*n* = 15)	FD patients with normal levels of acylated ghrelin (*n* = 33)	FD patients with low levels of acylated ghrelin (*n* = 35)
STAI state	47.9 ± 6.59	57 ± 4.47	46.7 ± 4.44
STAI trait	47.3 ± 6.56	45.6 ± 4.76	35.3 ± 4.67
SRQ-D	11.2 ± 1.34*	11.8 ± 0.94**	8.23 ± 0.72
PCS	42.4 ± 1.96***	41.2 ± 1.41****	47.6 ± 0.88
MCS	47.5 ± 1.33	46.2 ± 1.22	48.1 ± 0.94
PSQI	4.86 ± 0.42*****	7.55 ± 0.63******	5.46 ± 0.61

*p = 0.040, vs FD patients with low levels of acylated ghrelin; **p = 0.0035 vs FD patients with low levels of acylated ghrelin; ***p = 0.0076, vs FD patients with low levels of acylated ghrelin; ****p<0.001, vs FD patients with low levels of acylated ghrelin; *****p = 0.0071, vs FD patients with normal levels of acylated ghrelin; ******p = 0.020, FD patients with low levels of acylated ghrelin; STAI, State-Trait Anxiety Inventory; SRQ-D, Self-Rating Questionnaire for Depression; PCS, Physical Component Summary; MCS, Mental Component Summary; PSQI, Pittsburgh Sleep Quality Index.

**Table 3:** Comparison of gastric emptying with acylated ghrelin levels in FD patients

	FD patients with high levels of acylated ghrelin (*n* = 15)	FD patients with normal levels of acylated ghrelin (*n* = 33)	FD patients with low levels of acylated ghrelin (*n* = 35)
Tmax	58.8 ± 2.87	60.5 ± 5.5	65.9 ± 5.51
T_1/2_	92.5 ± 5.35	104.8 ± 14.4	113.9 ± 15.5
AUC_5_	22.4 ± 2.08	19.8 ± 1.27	18.4 ± 1.00
AUC_15_	51.7 ± 3.48	47.0 ± 2.53	44.8 ± 1.99

Tmax, The lag phase as the point of maximum gastric emptying; T_1/2_, half gastric emptying time; ACU_5_, area under the curve at 5 min; AUC_15_, area under the curve at 5 min.

**Table 4:** Relationship between endoscopic findings based on Kyoto classification and acylated ghrelin levels in FD patients

	FD patients with high levels of acylated ghrelin (*n* = 35)	FD patients with more than low levels of acylated ghrelin (*n* = 69)	*p* value
Gastric atrophy	1.63 ± 0.092*	1.01 ± 0.098	0.00011
Intestinal metaplasia	0.77 ± 0.15**	0.33 ± 0.084	0.0077
Enlarged fold	0 ± 0	0.029 ± 0.029	0.48
Swelling of area gastrica	0.14 ± 0.073	0.043 ± 0.032	0.15
Nodularity	0.029 ± 0.029	0.043 ± 0.025	0.71
Spotty redness	0.14 ± 0.083	0.029 ± 0.029	0.12
Diffuse redness	0.34 ± 0.13	0.12 ± 0.057	0.064
Xanthoma	0.31 ± 0.11***	0.10 ± 0.042	0.036
Hematin	0.17 ± 0.096	0.33 ± 0.089	0.26
Kammrotung	0 ± 0****	0.30 ± 0.083	0.011
Mucosal swelling	0 ± 0	0.014 ± 0.014	0.48
Patchy redness	0.14 ± 0.083	0.058 ± 0.041	0.30
Flat erosion	0.086 ± 0.063	0.12 ± 0.049	0.71
Mucus	0.23 ± 0.083^†^	0.029 ± 0.029	0.0063
Fundic gland polyp	0.2 ± 0.099	0.52 ± 0.10	0.051
Visibility of vascular pattern	0.57 ± 0.14	0.39 ± 0.088	0.26
RAC in angle	0.91 ± 0.17^††^	1.38 ± 0.11	0.022
Raised erosion	0.14 ± 0.073	0.13 ± 0.046	0.88

*p<0.001, vs FD patients with more than low levels of acylated ghrelin (compared group); **p = 0.0077 vs compared group; ***p = 0.036, vs compared group; ****p = 0.011, vs compared group; ^†^*p* = 0.0063, vs compared group; ^††^*p* = 0.022, vs compared group.

## References

[1] Kojima M, Hosoda H, Date Y, Nakazato M, Matsuo H, Kangawa K Ghrelin is a growth-hormone-releasing acylated peptide from stomach. Nature 1999; 402: 656–660. 10604470 10.1038/45230

[2] Cummings DE Gherelin and the short-and long-term regulatation of appetite and body weight. Physiol Behav 2006; 89: 71–84. 16859720 10.1016/j.physbeh.2006.05.022

[3] Quigley EM Gastric motor and sensory function and motor disorders of the stomach. In: Feldman M, Friedman LS, Sleisenger, eds Gastrointestinal and Liver Disease Pathophysiology/Diagnosis/Management. Philadelphia: WB Saunders, 2002; 691–714.

[4] Dixi VD, Taub DD Gherelin and immunity: a young player in an old field. Exp Gerontol 2005; 40: 900–910. 16233968 10.1016/j.exger.2005.09.003

[5] Gonzalez-Rey E, Chorny A, Delgado M Therapeutic action of ghrelin in a mouse model of colitis. Gastroenterology 2006; 130: 1707–1720. 16697735 10.1053/j.gastro.2006.01.041

[6] Ziko I, Sominsky L, De Luca SN, Lelngei F, Spencer SJ Acylated ghrelin suppresses the cytokine response to lipopolysaccharide and does so independently of the hypothalamic-pituitary-adrenal axis. Brain Behav Immun 2018; 74: 86–95. 30009998 10.1016/j.bbi.2018.07.011

[7] Shiiya T, Nakazato M, Mizuta M, et al Plasma ghrelin levels in lean and obese humans and the effect of glucose on ghrelin secretion. J Clin Endocrinol Metab 2002; 87: 240–244. 11788653 10.1210/jcem.87.1.8129

[8] Ulasoglu C, Isbilen B, Doganay L, Ozen F, Kizitas S, Tuncer I Effect of *Helicobacter pylori* eradication on serum ghrelin and obestatin levels. World J Gastroenterol 2013; 19: 2388–2394. 23613634 10.3748/wjg.v19.i15.2388 PMC3631992

[9] Nweneka CV, Prentice AM *Helicobacter pylori* infection and circulating ghrelin levels - a systematic review. BMC Gastroenterol 2011; 11: 7. 21269467 10.1186/1471-230X-11-7 PMC3037919

[10] Liew PL, Lee WJ, Lee YC, Chen WY Gastric ghrelin expression associated with Helicobacter pylori infection and chronic gastritis in obese patients. Obes Surg 2006; 16: 612–619. 16687031 10.1381/096089206776945002

[11] Sugano K, Tack J, Kuipers EJ, et al Kyoto global consensus report on *Helicobacter pylori* gastritis. Gut 2015; 64: 1353–1367. 26187502 10.1136/gutjnl-2015-309252 PMC4552923

[12] Nomura S, Ida K, Terao S, et al.; Research Group for Establishment of Endoscopic Diagnosis of Chronic Gastritis Endoscopic diagnosis of gastric mucosal atrophy: multicenter prospective study. Dig Endosc 2014; 26: 709–719. 24698334 10.1111/den.12286

[13] Capelle LG, de Vries AC, Haringsma J, et al The staging of gastritis with the OLGA system by using intestinal metaplasia as an accurate alternative for atrophic gastritis. Gastrointest Endosc 2010; 71: 1150–1158. 20381801 10.1016/j.gie.2009.12.029

[14] Masuyama H, Yoshitake N, Sasai T, et al Relationship between the degree of endoscopic atrophy of the gastric mucosa and carcinogenic risk. Digestion 2015; 91: 30–36. 25632914 10.1159/000368807 PMC5348729

[15] Shindo T, Futagami S, Hiratsuka, et al Comparison of gastric emptying and plasma ghrelin levels in patients with functional dyspepsia and non-erosive reflux disease. Digestion 2009; 79: 65–72. 19246923 10.1159/000205740

[16] Chuang JC, Zigman JM Ghrelin’s roles in stress, mood, and anxiety regulation. Int J Pept 2010. DOI: 10.1155/2010/460549 10.1155/2010/460549 PMC2915752 20721341

[17] Lutter M, Sakata I, Osborne-Lawrence S, et al The orexigenic hormone ghrelin defends against depressive symptoms of chronic stress. Nat Neurosci 2008; 11: 752–753. 18552842 10.1038/nn.2139 PMC2765052

[18] Kim SH, Kim JW, Byun J, Jeong JB, Kim BG, Lee KL Plasma ghrelin level and plasma ghrelin/obestatin ratio are related to intestinal metaplasia in elderly patients with functional dyspepsia. PLoS One 2017; 12: e0175231. 28419119 10.1371/journal.pone.0175231 PMC5395142

[19] Drossman DA The functional gastrointestinal disorders and the Rome III process. Gastroenterology 2006; 130: 1377–1390. 16678553 10.1053/j.gastro.2006.03.008

[20] Svedlund J, Sjödin I, Dotevall G GSRS--a clinical rating scale for gastrointestinal symptoms in patients with irritable bowel syndrome and peptic ulcer disease. Dig Dis Sci 1988; 33: 129–134. 3123181 10.1007/BF01535722

[21] Spielberger CD, Goursuch RL, Lushine R Manual for the State-Trait Anxiety Inventory. White Bear Lake: Consulting Psychologists Press, 1983.

[22] Rockliff BW A brief self-rating questionnaire for depression (SRQ-D). Psychosomatics 1969; 10: 235–243. 10.1016/S0033-3182(69)71734-0 5808615

[23] Fukuhara S, Suzukamo Y Manual of the SF-8 Japanese Version. Kyoto: Institute for Health Outcomes & Process Evaluation Research, 2004.

[24] Doi Y, Minowa M, Okawa M, Uchiyama M Development of the Japanese version of the Pittsburgh Sleep Quality Index. Jpn J Psychiatry Treatment 1998; 13: 755–763.

[25] Doi Y, Minowa M, Okawa M, Uchiyama M Prevalence of sleep disturbance and hypnotic medication use in relation to sociodemographic factors in the general Japanese adult population. J Epidemiol 2000; 10: 79–86. 10778031 10.2188/jea.10.79

[26] Hellmig S, Von Schöning, Gadow C, et al Gastric emptying time of fluids and solids in healthy subjects determined by ^13^C breath tests: influence of age, sex and body mass index. J Gastroenterol Hepatol 2006; 21: 1832–1838. 17074022 10.1111/j.1440-1746.2006.04449.x

[27] Futagami S, Shindo T, Kawagoe T, et al Migration of eosinophils and CCR2-/CD68-double positive cells into the duodenal mucosa of patients with postinfectious functional dyspepsia. Am J Gastroenterol 2010; 105: 1835–1842. 20461070 10.1038/ajg.2010.151

[28] Nishizawa T, Suzuki H, Nomoto Y, et al Enhanced plasma ghrelin levels in patients with functional dyspepsia. Aliment Pharmacol Ther 2006; 2: 104–110.

[29] Lee KJ, Vos R, Janssens J, Tack J Influence of duodenal acidification on the sensorimotor function of the proximal stomach in humans. Am J Physiol Gastrointest Liver Physiol 2004; 286: G278–G284. 12760903 10.1152/ajpgi.00086.2003

[30] Algul S, Ozcelik O Evaluating the levels of nesfatin-1 and ghrelin hormones in patients with moderate and severe major depressive disorders. Psychiatry Investig 2018; 15: 214–218. 10.30773/pi.2017.05.24 PMC5900400 29475222

[31] Jerlhag E, Egecioglu E, Landgren S, et al Requirement of central ghrelin signaling for alcohol reward. Proc Natl Acad Sci U S A 2009; 106: 11318–11323. 19564604 10.1073/pnas.0812809106 PMC2703665

[32] Kluge M, Gazea M, Schüssler P, et al Ghrelin increases slow wave sleep and stage 2 sleep and decreases stage 1 sleep and REM sleep in elderly men but does not affect sleep in elderly women. Psychoneuroendocrinology 2010; 35: 297–304. 19647945 10.1016/j.psyneuen.2009.07.007

[33] Shinomiya T, Fukunaga M, Akamizu T, et al Plasma acylated ghrelin levels correlate with subjective symptoms of functional dyspepsia in female patients. Scand J Gastroenterol 2005; 40: 648–653. 16036524 10.1080/00365520510015403

[34] Yamawaki H, Futagami S, Shimpuku M, et al Leu72Met408 polymorphism of the ghrelin gene is associated with early phase of gastric emptying in the patients with functional dyspepsia in Japan. J Neurogastroenterol Motil 2015; 21: 93–102. 25540946 10.5056/jnm14086 PMC4288091

[35] Khoo J, Rayner CK, Fenile-Bisset C, Jones KL, Horowitz M Gastrointestinal hormonal dysfunction in gastroparesis and functional dyspepsia. Neurogastroenterol Motil 2010; 22: 1270–1278. 20939851 10.1111/j.1365-2982.2010.01609.x

[36] Shimpuku M, Futagami S, Inamori M, et al Distinct associations between depression status and initial phase of gastric emptying in functional dyspepsia and healthy volunteers. Int J gastroenterol Disord Ther 2014; 1: 106–111.

[37] Suzuki H, Masaoka T, Hosoda H, et al Plasma ghrelin concentration correlates with the levels of serum pepsinogen I and pepsinogen I/II ratio--a possible novel and non-invasive marker for gastric atrophy. Hepatogastroenterology 2004; 51: 1249–1254. 15362725

[38] Gao XY, Kuang HY, Liu XM, Ma ZB, Nie HJ, Guo H Plasma obestatin levels in men with chronic atrophic gastritis. Peptides 2008; 29: 1749–1754. 18588931 10.1016/j.peptides.2008.05.027

